# Prophylaxis against Asphyxiating Gases

**Published:** 1916-10

**Authors:** 


**Prophylaxis Against Asphyxiating Gases.** Peyronnet of Lafonville, Jour. de Med. et Chir. Prat., Jan. 10, states that chlorin is the principal gas used by the Germans and that English officers who discarded their masks to issue commands were almost immediately suffocated. A wad of cotton in gauze is saturated with a solution of sodium hyposulphite 1,000, sodium carbonate 200, glycerin 150, and water 800.

